# Polydisperse Aerosol Transport and Deposition in Upper Airways of Age-Specific Lung

**DOI:** 10.3390/ijerph18126239

**Published:** 2021-06-09

**Authors:** Mohammad S. Islam, Puchanee Larpruenrudee, Sheikh I. Hossain, Mohammad Rahimi-Gorji, Yuantong Gu, Suvash C. Saha, Gunther Paul

**Affiliations:** 1 School of Mechanical and Mechatronic Engineering, University of Technology Sydney (UTS), 15 Broadway, Ultimo, NSW 2007, Australia; Puchanee.Larpruenrudee@uts.edu.au; 2 School of Life Sciences, University of Technology Sydney, Ultimo, NSW 2007, Australia; SheikhImamul.Hossain@uts.edu.au; 3 Department of Human Structure and Repair, Ghent University, 9000 Ghent, Belgium; mohammad.rahimigorji@ugent.be; 4 School of Mechanical, Medical and Process Engineering, Faculty of Engineering, Queensland University of Technology, Brisbane, QLD 4000, Australia; yuantong.gu@qut.edu.au; 5 Australian Institute of Tropical Health and Medicine, James Cook University, Townsville, QLD 4810, Australia; gunther.paul@jcu.edu.au

**Keywords:** airway reduction, aging, particle transport, LES, drug-aerosol delivery

## Abstract

A comprehensive understanding of airflow characteristics and particle transport in the human lung can be useful in modelling to inform clinical diagnosis, treatment, and management, including prescription medication and risk assessment for rehabilitation. One of the difficulties in clinical treatment of lung disorders lies in the patients’ variable physical lung characteristics caused by age, amongst other factors, such as different lung sizes. A precise understanding of the comparison between different age groups with various flow rates is missing in the literature, and this study aims to analyse the airflow and aerosol transport within the age-specific lung. ANSYS Fluent solver and the large-eddy simulation (LES) model were employed for the numerical simulation. The numerical model was validated with the available literature and the computational results showed airway size-reduction significantly affected airflow and particle transport in the upper airways. This study reports higher deposition at the mouth-throat region for larger diameter particles. The overall deposition efficiency (DE) increased with airway size reduction and flow rate. Lung aging effected the pressure distribution and a higher pressure drop was reported for the aged lung as compared to the younger lung. These findings could inform medical management through individualised simulation of drug-aerosol delivery processes for the patient-specific lung.

## 1. Introduction

The primary sources of airborne particles are various natural events and manufacturing activities. The excessive use of vehicles, occurrence of bush-fires, home cooking, various manufacturing processes, complex chemical reactions, and nanomaterials industrialisation expose a substantial amount of particles to the air [1,2]. The atmospheric air consists of polycyclic aromatic hydrocarbons (PAHs) (particulate and gaseous), which are created from the incomplete burning of organic materials [3]. Atmospheric aerosols and PAHs are complex in terms of size distribution. It was reported that the aerosol size with 50% penetration for the respirable fraction is 4 µm in diameter [4]. The respirable particle can travel to the deeper airways due to the small size fraction [5] and, depending on the residence time, chemical composition, and toxicity, the inhaled particle can damage the respiratory system of children [6,7] and older adults [3,8]. Smaller diameter aerosols can travel to the lower airways, penetrating the airway’s mucus layer, and may be soluble in the epithelium cells [9]. Some insoluble aerosols can damage the epithelium cells, reduce lung function, produce various respiratory diseases, and enter the body’s bloodstream [10]. The precise knowledge of aerosol transport within the human respiratory tract plays a significant role in medical treatment, as it enables the prediction of drug delivery performance in the lungs [11,12]. In general, respiratory disorders are preferably treated by transferring drugs to patients through inhalation [13,14,15]. To administer a dose and assess the risk from inhaled aerosols, the prediction of particle deposition is important for the treatment of respiratory ailments [16,17]. Particle deposition is usually based on various factors such as age, flow rate, particle size, the physical and chemical properties of particles, and the respiratory tract geometry. In the human body, internal organs normally grow with body size and develop from infancy to adulthood. These changes include lung volume, alveoli size, lung size, and other parts of the respiratory tract [18,19]. Therefore, the understanding of particle deposition in the human airways requires consideration of different lung characteristics which are age-dependent. 

Particle deposition in the respiratory tract is estimated by various methods. The specific parts of the respiratory tract that have been studied are the nasal airways [20,21,22,23], mouth-throat area [24,25,26,27], and tracheobronchial airways [28,29,30,31,32,33]. Age effects on particle deposition in the human lungs have been estimated, in earlier studies, using theoretical models and morphometric measurement [16,28,34,35,36,37,38]. Moreover, even recent studies have used the same methods [39,40]. These studies have mainly focused on particle deposition within the lungs through the comparison of infant, child, adolescent, and adult cases. They all agreed that the deposition efficiency was higher in younger people, specifically in the higher generations of the tracheobronchial airways, as compared to older people. 

Most recently, the airflow characteristics of human lungs have been studied using computational fluid dynamics (CFD) models, which allow for the comparison of different lung sizes as a function of aging [33,41,42]. Bass et al. performed CFD and in vitro analysis on three age groups of children (2–3 years, 5–6 years, and 9–10 years) [43]. The study reported that airway dimensions could affect respiratory dynamics, and the study considered airways up to the first two bifurcations. Vinchurkar et al. [44] studied drug deposition of inhaled medication in five asthmatic patients, aged between 38 to 63 years, for the upper airways, and reported lower deposition with lungs of high extrathoracic resistance. The multiple path particle dosimetry (MPPD) model did not present the deposition pattern for patient-specific cases. Katan et al. [45] studied inhalation therapy in the idealised acinar region and showed aerosol size affects on the deposition pattern. The anatomy of the acinus model was far from the real geometry. Deng et al. [46] studied microparticle deposition in the tracheobronchial airways using Weibel’s model of airways in infants, children, and adults. Their models consisted of upper airways and lower airways. The results of the study showed different particle deposition rates as a function of aging and different airway geometry. Das et al. [47] investigated micron particle delivery to the upper airways of the child and adult lung. The non-realistic airway model reported different deposition patterns for various aerosol sizes. Manojkumar et al. [48] investigated the deposition of particulate matter (PM) as PM1, PM2.5, and PM10 in human lungs for the age groups of infants, children, and adults. The analytical MPPD model concluded that children have the highest PM deposition rate, followed by adults and, lastly, infants. Furthermore, PM2.5 was considered the most important size fraction deposited in the lungs. 

All of the available in silico and in vitro studies investigated the fluid flow and particle transport for different age groups and improved the knowledge of the field. However, no comprehensive numerical or experimental study investigated the aerosol transport to the combined mouth-throat and upper airways for people over 50-years-old. This study aims to analyse the airflow and particle transport to the mouth-throat and upper airways of a 50-, 60-, and 70-year-old human lung for the first time. In reality, aerosol size and composition could change inside the respiratory system at high temperature and humid conditions [49]. This study did not consider any condensation effects of the aerosols. 

## 2. Methods

### 2.1. Geometrical Development

**Figure 1:**
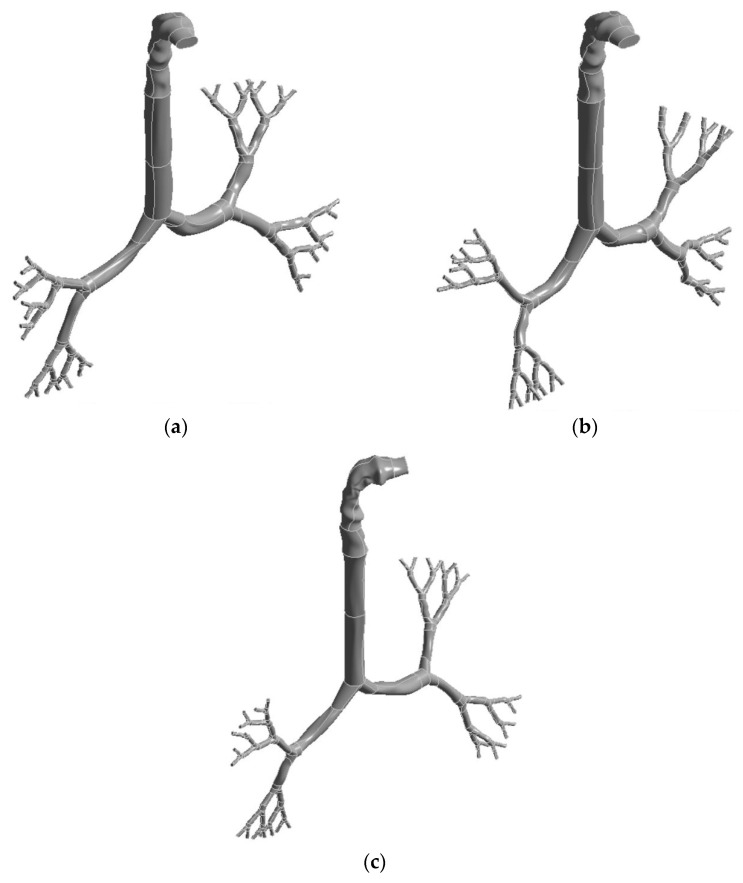
Construction of models from a CT-based mouth-throat model and Weibel’s model of a large bronchial airway: (**a**) 50-year-old lung, (**b**) 60-year-old lung, and (**c**) 70-year-old lung.

Three models were generated for the 50-, 60-, and 70-year-old lung by combining the CT-based upper airway model with the scalable bronchial airway model. While all three models contained the same CT-based upper airway model, the size of the large bronchial airway, beginning from the trachea to five generations, was different for all three cases. Niewoehner et al. [50] analysed the aging effects and airway resistance for the human lung. The study reported a 10% reduction in the airway size. Later, Xu and Yu [16] developed an analytical equation for the age-specific lung based on this finding. Recently, Kim et al. [41] performed a CFD study and developed an age-specific anatomical model. The study used the 10% reduction of the diameter for aged lungs over 50 years old. For a 50-year-old lung, we employed the dimensions from Weibel’s model from the trachea to generation 5. We then improved on the first model and made it more realistic by making the airways more resilient with uneven surfaces. For the 60- and 70-year-old lung models, the dimensions from the trachea to generation 5 were reduced by 10% and 20%, respectively. Figure 1 shows the reconstructed anatomical model for the three different bronchial airway dimensions. Figure 1a represents the reconstructed bronchial airway for a 50-year-old, while Figure 1b,c represent the 60- and 70-year-old airways, respectively.

### 2.2. Grid Generation and Model Validation

**Figure 2:**
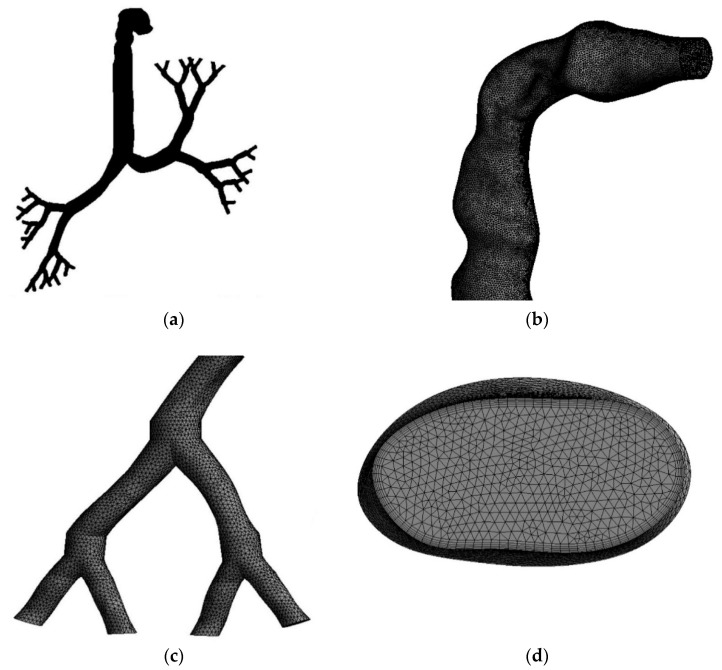
Unstructured mesh for selected sections of the airway model; (**a**) mesh for the whole lung model (cells invisible because of a large number of small cells), (**b**) tetrahedral elements for the mouth-throat section, (**c**) the mesh at a bifurcation branch, and (**d**) inflation mesh at the inlet cross-section of the mouth area.

The mesh for the whole model, which includes the highly asymmetric mouth-throat and large bronchial airways, was created with the ANSYS meshing-module using unstructured tetrahedral elements. Figure 2 presents the mesh in different parts of the model. Figure 2a shows the whole lung model with tetrahedral elements. Figure 2b,c magnify the specific areas of the mouth-throat and the fifth bifurcation of the airways, respectively. An inflation layer mesh was generated for the whole lung model to avoid contradicting spaces, control particle movement inside the lungs, and compensate for irregular or bend shapes (shown in Figure 2d). 

**Figure 3:**
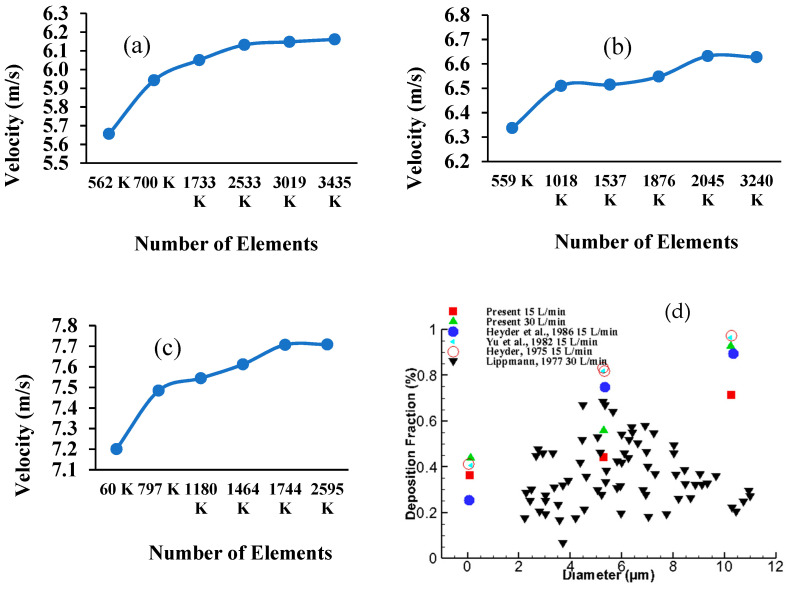
Mesh independence test results for (**a**) 50-year-old model, (**b**) 60-year-old model, (**c**) 70-year-old model, and (**d**) deposition fraction comparison with available experimental data [51,52,53,54].

The body sizing method was used for mesh refinement in order to generate mesh cells with a good orthogonal quality, using all five layers of inflation. As the three lung models have different dimensions, grid refinement was applied to all three models with different adjustment parameters. The different meshes were tested under the maximum velocity criterion at a selected plane of the mouth-throat area. Figure 3 shows grid independence for all three models. The velocity was found to be stable at 2.5 million elements for the 50-year-old model, Figure 3a, while stable velocities for the 60-year-old model and 70-year-old model were found at 2.0 million and 1.7 million elements, respectively (Figure 3b,c). Figure 3d shows the deposition fraction comparison at the mouth-throat area with the available literature [51,52,53,54]. The deposition fraction value from the present simulation are in the range of available experimental measurement. 

### 2.3. Numerical Methods

As mentioned previously, the mouth-throat area for all models was taken from a CT scan, while other sections, including the trachea and the first 5 generations, were reconstructed. This study presents airflow patterns, especially particle transport and deposition caused by airway reduction, as a function of age.

The fluid flowfield was calculated by using the mass equation (Equation (1)) and the momentum equation (Equation (2)) [32]:

(1) $$\frac{\partial \rho }{\partial t}+\nabla \cdot \left(\rho \vec{v}\right)=0$$

(2) $$\frac{\partial }{\partial t}\left(\rho \vec{v}\right)+\nabla \cdot \left(\rho \vec{v}\vec{v}\right)=-\nabla \rho +\nabla \cdot \left(\mu \left[\left(\nabla \vec{v}+\nabla {\vec{v}}^{T}\right)-\frac{2}{3}\nabla \cdot \vec{v}I\right]\right)+\rho \vec{g}+\vec{F}$$

where, ρ is the fluid static pressure, $\rho \vec{g}$ is a body force from gravity, and $\vec{F}$ is a body force from external forces. 

The large-eddy simulation (LES) model was used in order to calculate the airflow [55]. Under the LES model, a Smagorinsky-Lilly subgrid scale (SGS) model was used to calculate the rest of the smaller eddies. Pressure-velocity coupling was used with a simple scheme, second-order pressure, and second-order upwind for momentum. Particle transport was calculated using a continuous fluid Lagrangian discrete phase model (DPM), as expressed in the following equation:

(3) $${\vec{F}}_{D,i}=\frac{1}{2}C_{D}\frac{\pi d_{\rho ,i}^{2}}{4}\rho \left({\vec{v}}_{\rho ,i}-\vec{v}\right)\left|{\vec{v}}_{\rho ,i}-\vec{v}\right|$$

where, $C_{D}$ is the drag coefficient, $d_{p}$ is the particle diameter, and ${\vec{v}}_{\rho }$ is the particle velocity. In this study, Newton’s second law was employed to calculate the individual particle i motion:

(4) $$m_{\rho ,i}\frac{\partial {\vec{v}}_{\rho ,i}}{\partial t}={\vec{F}}_{D,i}+m_{\rho ,i}\vec{g}$$

and particle position:

(5) $$\frac{\partial x_{p}}{\partial t}={\vec{v}}_{p}$$

Under the DPM model, the surface injection method was used to release the particles to all parts of the lung models. The particles were set to be injected from the inlet surface at the mouth area through other sections of the lung airways. The boundary conditions were taken as the inlet velocity at the mouth area and the outlet outflow at the end of the fifth generation. The inlet velocities were based on four different flow rates representing daily physical activities, which were 7.5 L/min, 15 L/min, 30 L/min, and 60 L/min. The pressure was set as a zero-gauge pressure condition. The physical input properties are presented in Table 1.

The Rosin-Rammler method was applied to introduce the polydisperse aerosol particles, which ranged in size from 1 µm to 10 µm. The average total particle counts per injection, irrespective of size, was 12,500 particles for all three cases. We used non-uniform size distribution and spherical particles. A “trap” condition was used, as particles can be trapped when touching the walls, which contain sticky mucus. The wall boundary was set to be a stationary wall, with a no-slip condition, which can lead the particle to become attached to the wall. The residual criterion was defined as a convergence of the continuity and momentum equations to a maximum of 0.00001.

## 3. Results

The three different lung models for different ages were simulated under various flow rates, simulating a low flow rate and a heavy flow rate based on daily physical activities. The simulation involved the inhalation of polydisperse aerosol particles in order to investigate the airflow and particle transport from the oral area through the upper airways and bronchial airways.

### 3.1. Velocity Contours 

**Figure 4:**
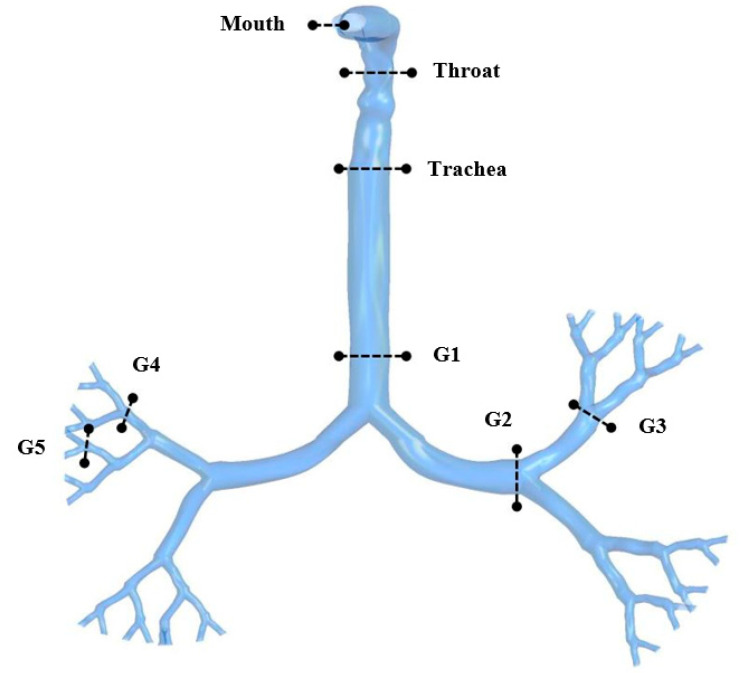
Selected planes for velocity contours at selected positions of the upper airways.

**Figure 5:**
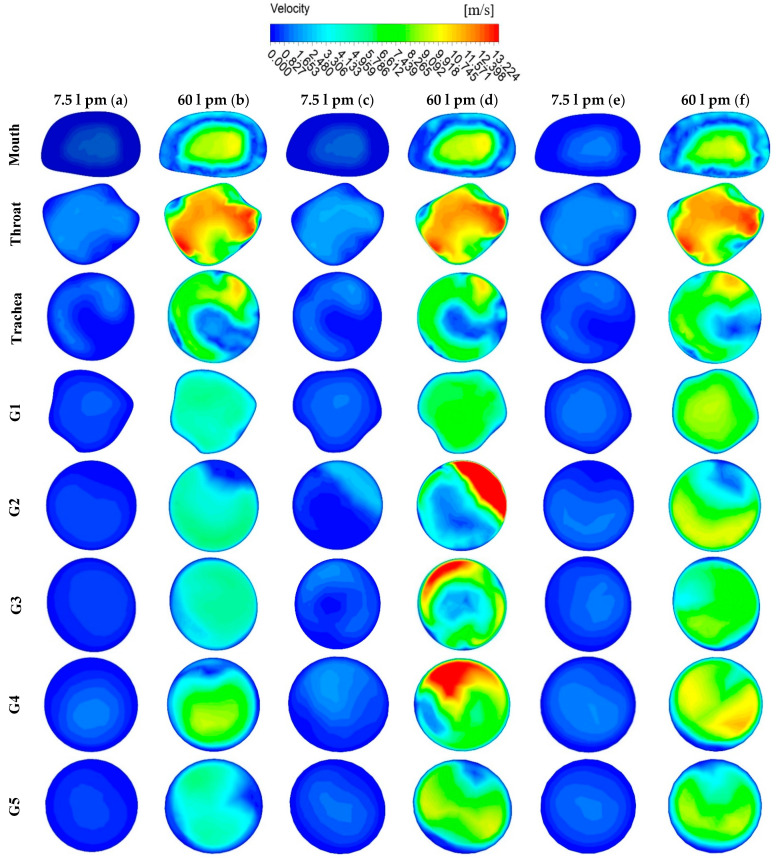
Velocity contours at selected positions of the upper airways, (**a**) 50-year-old, 7.5 L/min, (**b**) 50-year-old, 60 L/min, (**c**) 60-year-old, L/min, (**d**) 60-year-old, 60 L/min, (**e**) 70-year-old, 7.5 L/min, and (**f**) 70-year-old, 60 L/min. G1, Generation 1; G2, Generation 2; G3, Generation 3; G4, Generation 4; and G5, Generation 5.

Figure 4 presents the locations of plane selections in different areas of the whole lung model, starting from the oral area through generation 1 to generation 5. Figure 5 shows the velocity contours at selected positions from the mouth-throat area to generation 5 of the lungs for the three different lung dimensions. Figure 5a illustrates the 50-year-old lung velocity contours at a flow rate of 7.5 L/min, while Figure 5b is for 60 L/min. Figure 5c,d represents the contours for a 60-year-old lung at 7.5 L/min and 60 L/min, respectively. The velocity contours for a 70-year-old lung at 7.5 L/min and 60 L/min are presented in Figure 5e,f.

The comparison between flow rates at 7.5 L/min and 60 L/min showed significantly different velocity contours for the lower flow rate and the higher flow rate in all three lung age groups. Although the magnitude of velocity is different for the two flow rates, the velocity patterns remained similar in all three lung sizes. The maximum velocity in the 50-year-old lung and the 70-year-old lung were found at the throat area and for a flow rate of 60 L/min. However, the maximum velocity for the 60-year-old lung was found at generation 2. Velocity patterns at the mouth, throat, trachea, and generation 1 were found to be similar in all three lung sizes. The 60-year-old lung, however, tended to have higher velocity magnitudes at generations 2, 3, and, 4 when compared to other areas and other lung sizes. We observed that velocity patterns changed depending on the shape and size of lung airways. 

**Figure 6:**
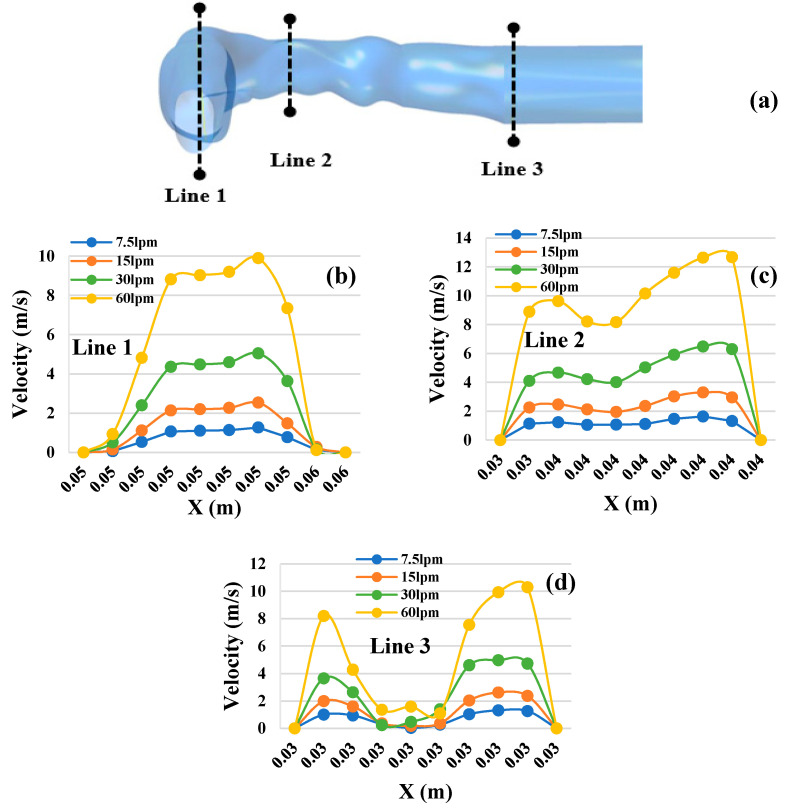

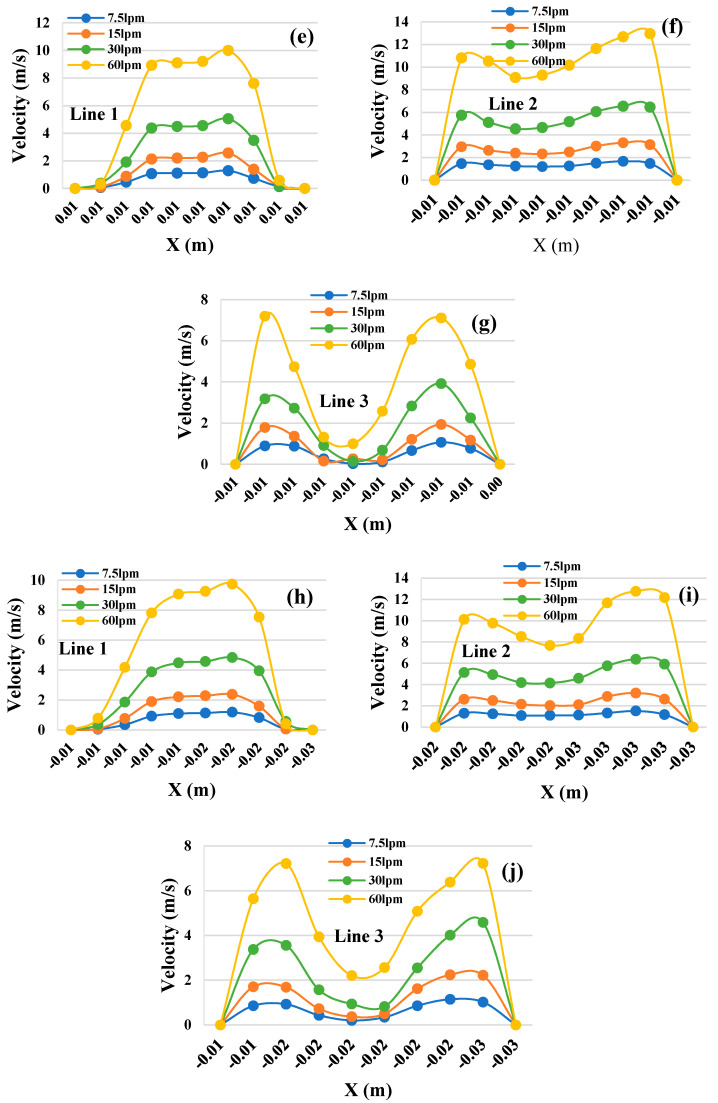
Velocity profiles for the three different lung sizes and for the four air flow rates at three selected cross-sections of the mouth-throat and trachea: (**a**) locations of cross-sections of the oral airways; velocity profile for 50-year-old lung (**b**) Line 1, (**c**) Line 2, (**d**) Line 3; velocity profile for 60-year-old lung (**e**) Line 1, (**f**) Line 2, (**g**) Line 3; velocity profile for 70-year-old lung (**h**) Line 1, (**i**) Line 2, and (**j**) Line 3.

To study the airflow patterns in the oral area, velocity profiles were calculated at selected positions of the mouth-throat and trachea areas. Figure 6 shows the velocity profiles at three selected cross-sections for the three different lung sizes and four different flow rates. Figure 6a shows the selected cross-sections of the airways in the oral area. Figure 6b–d show the velocity profiles in a 50-year-old lung, whereas Figure 6e–j show the velocity profiles for the 60- and 70-year-old lung. It can be seen in Figure 6 that the velocity profiles for each cross-section followed similar patterns in all three lung sizes. The throat area (Line 2) was found to have the highest velocity, while the lowest velocity was found at the trachea (Line 3) in all three cases. When comparing velocity patterns at the various flow rates, all flow rates generated similar velocity patterns for each area. It happened for all three lung cases. The highest velocities were found at 60 L/min at the throat cross-sections (Figure 6c,f,i) of all lung size cases. Velocities fluctuated most for the condition of 60 L/min flow rate at the trachea cross-section when compared to all other conditions. It is obvious that the throat area can experience higher velocities than other airway areas. 

### 3.2. Pressure Contours

**Figure 7:**
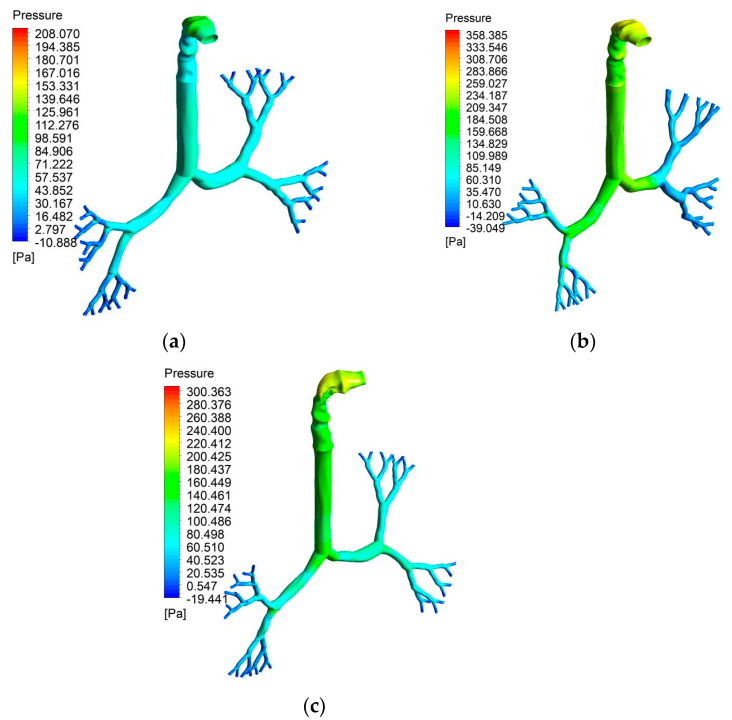
Pressure variation in the mouth-throat area and upper airways for three different lung sizes at 60 L/min flow rate: (**a**) 50-year-old lung, (**b**) 60-year-old lung, and (**c**) 70-year-old lung.

**Figure 8:**
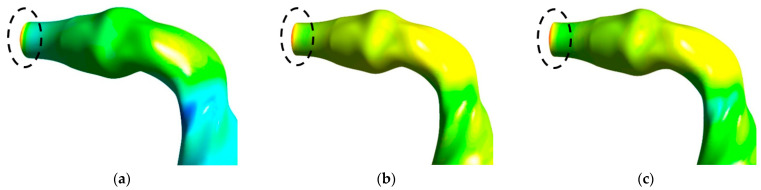
Pressure variation at the mouth-throat area: (**a**) 50-year-old lung, (**b**) 60-year-old lung, and (**c**) 70-year-old lung.

Figure 7 shows the pressure contours for all three lung sizes for a 60 L/min flow rate. Figure 7a presents pressure contours in the 50-year-old lung, while Figure 7b,c illustrate the 60- and 70-year-old lung. Figure 8 shows the pressure variation at the mouth-throat area for all three lung sizes for a 60 L/min flow rate. Figure 8a presents pressure contours in the 50-year-old lung, where Figure 8b,c presents the 60- and 70-year-old lung.

From Figure 7, it can be seen that the pressure generally decreases from the mouth-throat area to generation 5, which, in this study, was the lowest generation modelled in all three lung sizes. In addition, if focusing on the mouth-throat area from Figure 8, it is obvious that the highest pressure is indicated at the inlet of the mouth area for all three lung sizes (Figure 8a–c). 

**Figure 9:**
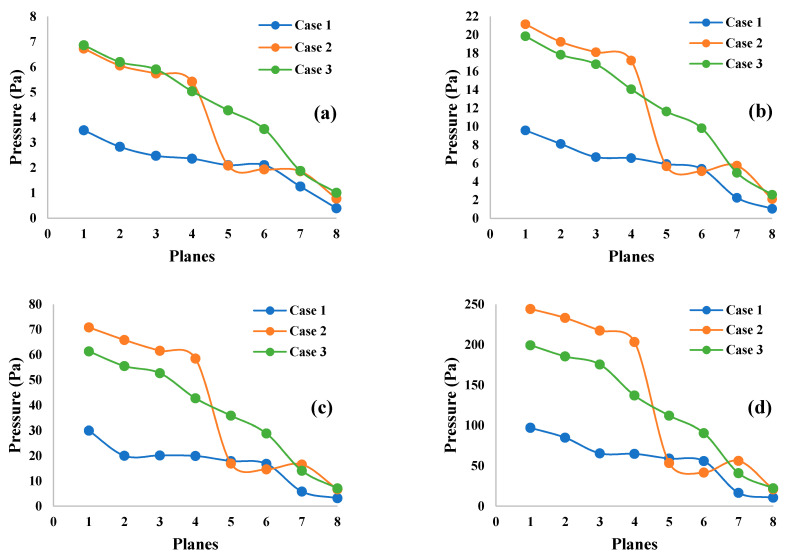
Pressure at selected positions of the mouth-throat and upper airways for three different lungs at (**a**) 7.5 L/min, (**b**) 15 L/min, (**c**) 30 L/min, and (**d**) 60 L/min. Case 1, 50-year-old lung; Case 2, 60-year-old lung; and Case 3, 70-year-old lung.

Pressures at different selected positions throughout the mouth-throat and upper airways are provided in Figure 9. Eight cross-section planes at different positions range from the mouth area (Plane 1), throat area (Plane 2), trachea area (Plane 3), and generation 1 to generation 5 (Plane 4 to Plane 8, respectively). Figure 9a,b present the pressures at 7.5 L/min and 15 L/min flow rates, whereas Figure 9c,d show the pressures at 30 L/min and 60 L/min flow rates. The maximum pressure always occurred in the mouth-throat area (Plane 1) for all flow rates. Subsequently, the pressure continued to decrease along the trachea (Plane 3) throughout the lower generations. The lowest pressure was located at generation 5 (Plane 8) for all flow rates. A lower flow rate always produces lower pressure, therefore a higher flow rate will result in higher pressure in all lung sizes. Pressures in the 50-year-old lung and the 70-year-old lung had similar pressure patterns that slowly declined for all flow rates, whereas the pressure pattern for the 60-year-old lung rapidly decreased for all flow rates from generation 1 (Plane 4) to generation 2 (Plane 5). The highly complex and asymmetric bifurcating structure affected the pressure variation from generation to generation for various ages. 

### 3.3. Particle Deposition Efficiency

In this study, particle DE was calculated based on the total numbers of particles injected into the lung airways under an inhaled condition from the mouth area. DE indicated the percentage of particles trapped during transportation through the lung airways, while the remaining particles escaped from generation 5 through to the lower generations.

**Figure 10:**
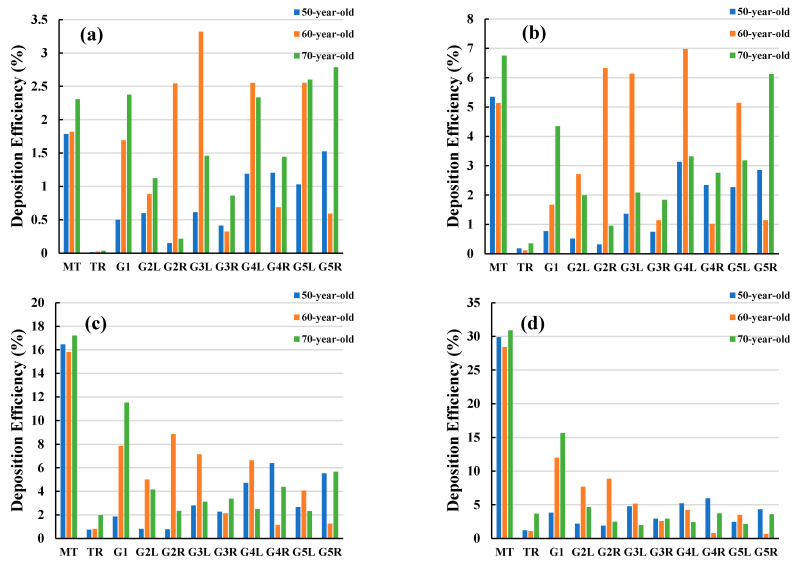
Polydisperse particle DE at various airway positions for different flow rates, (**a**) 7.5 L/min, (**b**) 15 L/min, (**c**) 30 L/min, and (**d**) 60 L/min. MT, mouth-throat; TR, trachea; G1, Generation 1; G2L, Generation 2-left lung; G2R, Generation 2-right lung; G3L, Generation 3-left lung; G3R, Generation 3-right lung; G4L, Generation 4-left lung; G4R, Generation 4-right lung; G5L, Generation 5-left lung; and G5R, Generation 5-right lung.

Figure 10 presents a comparison of DE at different airway positions for the three different ages at different flow rates. The DE patterns of flow rates 7.5 L/min and 15 L/min differ from the DE patterns at 30 L/min and 60 L/min. The overall DE showed an increasing trend with the flow rate. For the 50-year-old lung, the highest DE was found in the mouth-throat area for all flow rates. In the 60-year-old lung, the highest DE at 7.5 L/min and 15 L/min flow rates were found at generations 3 and 4. In the 70-year-old lung, the highest DE at 7.5 L/min and 15 L/min were located at generation 5 and in the mouth-throat area. For flow rates of 30 L/min and 60 L/min, the highest DE were again in the mouth-throat region for both the 60- and 70-year-old lungs. 

The irregularity of the complex shape in the mouth-throat area increases turbulence intensity, inertia, and air flow rate. These aspects possibly result in the higher DE in this area when compared to other areas [56]. The 70-year-old lung had the highest DE at 31% in the mouth-throat area at the highest flow rate, while the 50-year-old lung had the second highest DE at 30% and the 60-year-old lung had the lowest DE at 28%. When looking at the 50-year-old case at the highest flow rate, most particles were trapped at generation 4 after the dominant DE in the mouth-throat region. In the 60-year-old case, however, most particles were trapped at generation 3 after the dominant mouth-throat region. In the 70-year-old case, after the mouth-throat region, most particles were trapped at generation 1. DE at the mouth-throat area was different for all flow rates in the three lung models, as can be expected from thermodynamic considerations. More particles were transported from the first generation to the fifth generation, and escaped through lower generations at a lower flow rate.

**Figure 11:**
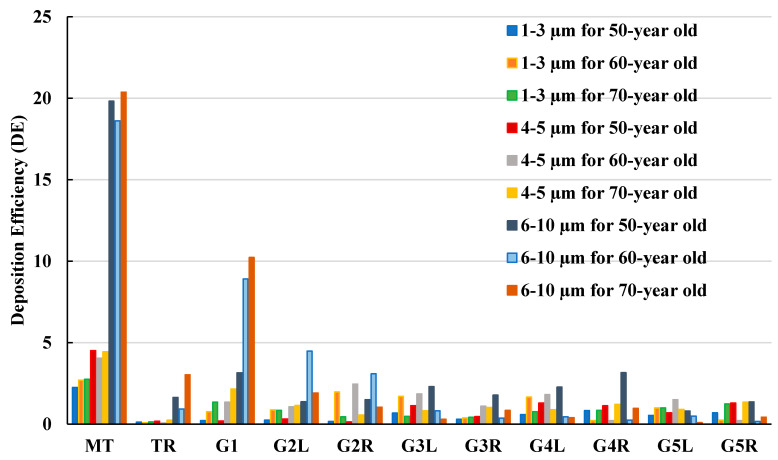
DE for different particle sizes at 60 L/m flow rate. MT, mouth-throat; TR, trachea; G1, Generation 1; G2L, Generation 2-left lung; G2R, Generation 2-right lung; G3L, Generation 3-left lung; G3R, Generation 3-right lung; G4L, Generation 4-left lung; G4R, Generation 4-right lung; G5L, Generation 5-left lung; and G5R, Generation 5-right lung.

Figure 11 shows particle deposition efficiency in selected areas of the lungs when inhaling. Particles were grouped into three classes of 1–3 µm, 4–5 µm, and 6–10 µm diameters, and DE was presented as a function of particle size. In all three lung size cases, most particles in the 6–10 µm range were deposited in the mouth-throat area. The highest DE was found in the 70-year-old lung with 20% DE and a similar DE was found in the other two lung cases. Generation 1 and the trachea were found to have the second and third highest DE for this particle size. Particles of 1–3 µm size were found to have the lowest DE for all lung size cases and in all regions. Thus, smaller particles of 1–3 µm will potentially escape through generation 5 to lower generations of the lungs, rather than larger particles which may be caught higher in the lungs.

**Figure 12:**
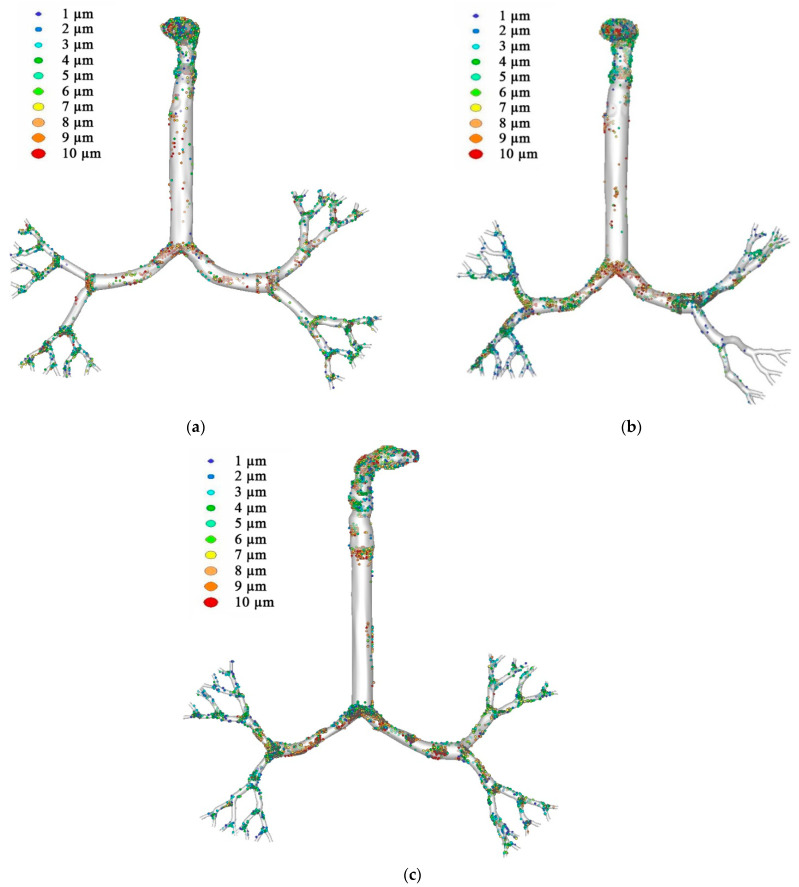
Aerosol deposition scenario at 60 L/min flow rate for (**a**) 50-year-old lung, (**b**) 60-year-old lung, and (**c**) 70-year-old lung. MT, mouth-throat; TR, trachea; G1, Generation 1; G2, Generation 2, G2R; G3 Generation 3; G4, Generation 4; and G5, Generation 5.

Figure 12 exemplifies an aerosol deposition scenario for the three different lung sizes at 60 L/min flow rate, where inhalation of aerosol particles occurred from the mouth through the airway, through to the trachea and upper generations. Figure 12a shows aerosol deposition in the 50-year-old lung, while Figure 12b,c show aerosol deposition in the 60- and 70-year-old lungs, respectively. Figure 12 is evidence that most inhaled particles are trapped in the mouth-throat area, however, particles settled in the bifurcations of all generations in all lung sizes.

## 4. Discussion

Numerical calculations were conducted based on realistic lung models from CT scans, which started from the mouth-throat through the upper airways and ended at generation 5. Due to the difference in lung geometries that have different sizes caused by age, the flow characteristics and pressures vary. The flow fields at the mouth-throat and bifurcating airways are found to be different for various ages of lung. The diameter reduction, due to lung aging and flow rate, influenced the velocity magnitude for all cases. The flow field became highly chaotic during the fast breathing condition (60 L/min), and the highest velocity magnitude was observed at the upper airways. The velocity profile at the upper part of the mouth-throat was fully developed, and the maximum velocity magnitude was observed at the centre of the mouth-throat. The lowest velocity was observed near the mouth-throat wall due to the no-slip condition. At the tracheal wall section, the flow field became highly complex and minimum velocity magnitude was reported at the centre of the tracheal wall. The velocity profile showed a maximum velocity magnitude near the walls of the tracheal airway. In some cases, and depending on disease manifestation, patients are treated using a mechanical ventilation apparatus. Using a ventilator may result in airway injury because of the high-pressure impact on the airways causing high resistance and stress. Therefore, the understanding of pressure variation in the lungs is expected to help improve the ventilation procedures of patients with severe respiratory diseases, such as asthma or COPD [1,57]. In terms of pressure variations, the highest pressure was found in the inlet of the mouth area in all three lung sizes studied. The 60-year-old lung generated the highest pressure. Lower generations of the lungs generated lower pressures in all lung sizes. The highest pressure occurred in the 60-year-old lung rather than the a 70-year-old lung, which had the smallest size. The lowest pressure occurred in the 50-year-old lung. Pressure dropped significantly between generation 1 and generation 2 in the 60-year-old lung.

The mouth-throat trapped more particles, especially in the 6–10 µm size range, resulting in higher DE. DE was significantly affected by flow rate and lung size. In the oral area, the smallest lung (70-year-old lung) had the highest DE followed by the 50-year-old lung and the 60-year-old lung for flow rates of 30 L/min and 60 L/min. Bifurcations of all generations trapped an exceeding number of particles in all lung sizes. This study proves that lung size has a considerable effect on particle transport and particle deposition within the human lung airways. Smaller lungs will trap more particles at all generations. We conclude that both flow rate and lung size significantly influence particle transport and particle deposition in the lungs. The mouth-throat area, with a higher flow rate, can trap most particles, whereas lower flow rates result in higher particle deposition at generations further down in the lungs. The overall deposition pattern also shows high concentrations at the carinal angle area of the airways. Microparticle inertia and inertial impaction are the dominant mechanisms of the deposition at the mouth-throat and upper airways. The anatomical shape of the present mouth-throat and upper airway model is highly asymmetric. The asymmetric shape of the airways also increases the overall deposition pattern. In normal flow condition, aerosol particle follows the air streamline. However, during inhalation and exhalation, when aerosol faces many bends and the uneven surface of the airway, the particle cannot follow the air streamline due to the inertia and deviates from the actual air path line. As a result, particles touch the airway wall. At the carinal angle area, the airway consists of a strong change of curvature. A larger diameter particle cannot follow the air streamline when it passes the carinal region and deposits on the bifurcation area. 

## 5. Conclusions

A simulation of airflow and particle transport in human lung airways was performed for three lung models. Three different lung models, with different dimensions from the trachea to the upper airways and different flow rates, were employed as the main factors in this study. The airway dimensions and flow rates were shown to significantly affect the flowfield at the mouth-throat and upper airways. At high inhalation, the flowfield became highly complex at the mouth-throat and tracheal area. The maximum pressure was observed for the 60-year-old lung at a high flow rate, while the 70-year-old model showed a maximum pressure at low rate. The overall DE increased with the flow rate and age. The mouth-throat and carinal angles were found to be the deposition hot spot for all cases. The results of this study could enhance the fundamental understanding of airflow characteristics, microparticle transport, and deposition within the human lung airways as they relate to lung aging. Future studies should consider more lung sizes and lung models which represent additional age-related factors, such as the dynamic properties of airway walls. Both environmental and occupational exposure to airborne hazardous pollutants is well established along with its pulmonary health effects [58,59]. Concerns are related to the associated human health risks as a response to the exposure to airborne pollutants, which vary by fraction of particulate matter, as well as their physical and chemical composition. Any model of a health response affecting the respiratory system must include and consider the most susceptible groups of the population, such as children and the elderly, when assessing environmental health outcomes.

### Limitations of the Study

- This study focused on airflow and particle transport in human lungs for an inhalation scenario;
- The model used only a small number of lung generations, where the end of generation 5 was set to be the outlet;
- The airway size for elderly patient-specific lung can increase, and this study did not consider this effect;
- The upper lobe was not considered for the airway model to make the similar geometry for all cases and minimise the reconstruction complexity;
- This study did not consider any dynamic wall motion or airway deformation;
- Open outlet conditions and constant pressure were used at the outlets.

## Figures and Tables

**Table 1:** Properties of air and aerosol.

Properties	Air	Aerosol
Density (kg/m^3^)	1.225	1100
Viscosity (kg/m-s)	1.7893 × 10^5^	-
